# Identification of two novel HSP90 proteins in *Babesia orientalis*: molecular characterization, and computational analyses of their structure, function, antigenicity and inhibitor interaction

**DOI:** 10.1186/1756-3305-7-293

**Published:** 2014-06-26

**Authors:** Muhammad Kasib Khan, Lan He, Weichao Zhang, Yifan Wang, Qing Tao, Qiqi Song, Muhammad Sohail Sajid, Qian Yu, Jinfang Hu, Rui Fang, Min Hu, Yanqin Zhou, Junlong Zhao

**Affiliations:** 1 State Key Laboratory of Agricultural Microbiology, College of Veterinary Medicine, Huazhong Agricultural University, Wuhan, Hubei 430070, China; 2 Key Laboratory of Animal Epidemical Disease and Infectious Zoonoses, Ministry of Agriculture, Huazhong Agricultural University, Hubei, Wuhan 430070, China; 3 Department of Parasitology, University of Agriculture, Faisalabad 38040, Pakistan

**Keywords:** *Babesia orientalis*, HSP90, Molecular characterization, Structure and functional analysis, Ligand docking, Antigenic epitopes

## Abstract

**Background:**

HSP90 protects the cells from heat stress and facilitates protein maturation and stability. The full genome sequences of piroplasms contain two putative HSP90 proteins, which are yet uncharacterized. To this end, the two putative HSP90 proteins of *Babesia orientalis* were identified and characterized by molecular and *in silico* methods.

**Methods:**

The two putative proteins in *B. orientalis* genome showing homology with putative HSP90 of other piroplasms were cloned and sequenced. A computational analysis was carried out to predict the antigenic determinants, structure and function of these proteins. The interactions of two HSP90 isoforms with respective inhibitors were also examined through docking analysis.

**Results:**

The length of BoHSP90-A gene (amplified from gDNA) was 2706 bp with one intron from position 997 to 1299 bp. This gene amplified from cDNA corresponded to full length CDS with an open reading frame (ORF) of 2403 bp encoding a 800 amino acid (AA) polypeptide with a predicted size of 91.02 kDa. The HSP90-B gene was intronless with an ORF of 2349 bp, and predicted polypeptide comprised of 797 AA with a size of 90.59 kDa. The AA sequences of these two proteins of *B. orientalis* were the most identical to those of *B. bovis*. The BoHSP90-A and BoHSP90-B were recognized as 90 kDa in the parasite lysate by the rabbit antisera raised against the recombinant BoHSP90 proteins. The anti-*B. orientalis* buffalo serum reacted with the rBoHSP90s expressed in *E. coli*, indicating that these proteins might be secreted by the parasite before entry into host cells. The overall structure and functional analyses showed several domains involved in ATPase activity, client protein binding and HSP90 dimerization. Likewise, several HSP90 inhibitors showed binding to ATP binding pockets of BoHSP90-A and BoHSP90-B, as observed through protein structure-ligand interaction analysis.

**Conclusions:**

The two putative HSP90 proteins in *B. orientalis* were recognized as 90 kDa. The rBoHSP90-A and rBoHSP90-B were reacted with the *B. orientalis* infected buffalo serum. The computational structure and functional analyses revealed that these two proteins may have chaperonic activity. The protein structure-ligand interaction analyses indicated that these two proteins had many drug target sites.

## Background

*Babesia orientalis* is a tick-borne intraerythrocytic protozoan parasite, which was identified as a new species in 1997 based on morphology, transmission and pathogenicity [1,2]. It was the phylogenetic analysis of *B. orientalis* based on 18S rRNA gene and Mitochondrial genome sequences that confirmed its taxonomic standing [3,4]. This pathogen is transmitted by *Rhipicephalus haemaphysaloides* and is known to cause babesiosis in water buffaloes [1,2]. The disease is endemic to most parts of central and southern China with reported cases of mortality [1,2,5]. The disease is mainly characterized by anemia, fever, icterus, hemoglobinuria and is often fatal in immunodeficient animals [3,4].

Heat shock protein 90 (HSP90) is one of the most abundant proteins in many cells and protects them from heat and oxidative stress by stabilizing proteins [6,7]. It also aids in the elimination of denatured and aggregated proteins that cannot function properly and may cause lethal damage to cells [8]. HSP90 is a key element of chaperone machinery under non-heat stress conditions and facilitates protein trafficking, maturation and stability [9]. The multichaperone complexes formed by HSP90 and co-chaperones determine the conformation of newly synthesized proteins, known as “client proteins” [10].

An 82 kDa protein of the HSP90 family has recently been identified in many protozoan parasites [11-15]. Several studies demonstrated that this HSP90 molecule is secreted in the milieu by extracellular infective forms of protozoa and is associated with the entry of parasite into the host cells [13,16]. Nevertheless, experimental evidence suggested that this molecule, localized both in cytosol and nucleus, is an essential component for stage differentiation and intracellular growth of many protozoans [11,16-19]. It is interesting to note that the full genome sequences of *Babesia* and *Theileria* also contain two HSP90 putative proteins, which have not been characterized yet (Additional file 1). To this end, the present study was conducted to identify and characterize the two novel HSP90 proteins in *B. orientalis*, including BoHSP90-A and BoHSP90-B. Their immunogenicity was assessed by reacting recombinant BoHSP90 proteins with anti-*B. orientalis* buffalo serum. The structure and functional analyses were performed through homology modeling. Various HSP90 inhibitors showing ligand interactions with BoHSP90-A and BoHSP90-B were identified through computer-based drug design.

## Methods

### *In silico* identification of two HSP90-like proteins of *B. orientalis*

The two putative *B. orientalis* HSP90 proteins were given the names “BoHSP90-A and BoHSP90-B”. The BoHSP90-A and BoHSP90-B were identified *in silico* from the full genome sequence of *B. orientalis* (unpublished sequence). Two putative HSP90 nucleotide sequences of *B. bovis* including BbHSP90 (XM_001611817.1) and BbHSP90 putative (XM_001610712.1) were obtained from GenBank using a BLAST search. The two *B. bovis* HSP90 sequences were aligned with *B. orientalis* genome sequence to find BoHSP90-A and BoHSP90-B gene sequences. The resulting sequences were confirmed through BLASTn search and multiple sequence alignment with all putative HSP90 genes of other apicomplexan parasites available in the GenBank.

### Parasites and animals

Two water buffaloes of ≥2 years old were purchased from a *Babesia* free area and used for the preparation of anti-*B. orientalis* serum. They were confirmed as clean for *B. orientalis* through reverse line blot hybridization [20]. The parasite was cultured in splenectomized buffalo by inoculating 4 ml of *B. orientalis* infected blood with 1% parasitaemia (Wuhan strain) according to He *et al.*[4]. The blood was collected everyday to monitor the parasitaemia until it reached 3%. The immune serum against *B. orientalis* from infected buffaloes was also isolated and stored at -20°C until further use.

Six Japanese white female rabbits (specific pathogen free, SPF) were used for the preparation of immune serum against rBoHSP90-A and rBoHSP90-B.

The animals used in all the experiments were housed and treated in accordance with the stipulated rules for the regulation of administration of affairs concerning laboratory animals of P.R. China. The animal protocols for these experiments were approved by Standing Committee of Hubei People’s Congress, P. R. China, Laboratory Animals Research Centre of Hubei province and the Ethics Committee of Huazhong Agricultural University (Permit number: 4200696657).

### Extraction of nucleic acids and preparation of cDNA

The blood samples from the jugular veins of experimentally infected buffaloes with 3% parasitaemia were collected in BD Vacutainer^®^ tubes containing EDTA (Qingdao Pharmacypro Co., Ltd.) for the extraction of DNA and RNA. The leukocytes were removed from the blood using Plasmodipur filters (EuroProxima, Arnhem, the Netherlands) and total RNA was extracted from 250 μl of RBCs using TRIzol^®^ RNA extraction kit (Invitrogen, USA) according to the manufacturer’s instruction. RNA samples were treated with DNase I (Invitrogen, USA); RNase inhibitor (RNaseOUT™ Recombinant Ribonuclease Inhibitor, Invitrogen, USA) was added and then the RNA was reverse transcribed using FastQuant^®^ RT Kit (TIANGEN Biotech (Beijing) Co., Ltd.) according to the manufacturer’s instruction.

The genomic DNA was extracted from 200 μl of infected blood using QIAamp^®^ DNA mini Kit (Qiagen, Hilden, Germany) following the manufacturer’s instructions and stored at -20 °C till further use.

### Amplification, cloning and sequencing of BoHSP90-A and BoHSP90-B genes

The oligonucleotide primers for the amplification of BoHSP90-A and BoHSP90-B genes were designed from full length sequences, as obtained from the genome sequence of *B. orientalis* through *in silico* method (Table 1). The two BoHSP90 genes were amplified from gDNA using Ex Taq^®^ Hot Start master mix (TaKaRa Biotechnology, Dalian, China). The thermalcycling parameters for both genes included: the activation of Taq polymerase at 95°C for 10 min followed by 35 cycles of denaturation at 95°C for 1 min, annealing at 60°C for 1 min and extension at 72°C for 3 min, and a final extension of 10 min at 72°C. The RNAse free water was used as a negative control.

**Table 1:** **Oligonucleotide primers used for the amplification and sequencing of *Babesia orientalis *** **HSP90-A and HSP90-B genes**

**Primer**	**Sequence (5′-3′)**	**Remarks**
***BoHSP90-A gene***		
1HSP90-F^a^	ATGACGGCTGGGGGAGCTCTG	Primers for amplification of ORF
1HSP90-R ^b^	TTAAGCAGCCACAGTGGGTGATTGC	
1HSP90F1^a^	CAAAACGAGCAGACCTTCCC	Primers for Sequencing
1HSP90F2^a^	CCCGAATGTGACGACTATTTGGA	
1HSP90F3^a^	CTTTTAAGGCTTATGATGACCC	
1HSP90F4^a^	GCGCTTGAACCTGTTGATGAG	
1HSP90R1 ^b^	GCCGTATCAAGACTTTCTGCCT	
1HSP90R2 ^b^	GAAACCAACACCGAACTGACC	
1HSP90R3 ^b^	TAACATGTTCCCATTCGTGACG	
1HSP90R4 ^b^	CGGGTTGTTTTGGCTTCATACG	
***BoHSP90-B gene***		
2HSP90-F^a^	ATGAAGCGGTCCATATTTTTC	Primers for amplification of ORF
2HSP90-R ^b^	TCAAAGTTCGTCATTGACGG	
2HSP90F1^a^	GGCTATTAGGATCAGGGTATCT	Primers for Sequencing
2HSP90F2^a^	TATCAGCGGTTCCCATTCAAAC	
2HSP90F3^a^	CAAGAAGAAGCTCCAGGAAGA	
2HSP90F4^a^	GAAGGATGACGATATGAACTC	
2HSP90R1^b^	TCCCGATTTGGCAATTGTT	
2HSP90R2 ^b^	TGTTTTCGATGCTCTCTTTCAT	
2HSP90R3 ^b^	CCCCAGTCAGAAACAACAACA	

Superscript a and b are indicating sense and antisense primers, respectively.

The resulting PCR products were electrophoresed using 2% ethidium bromide stained agarose gel and purified using the Universal DNA Purification Kit (TIANGEN Biotech (Beijing) Co., Ltd.) according to the manufacturer’s instructions. The purified products were cloned into pMD19-T vector (TaKaRa Biotechnology (Dalian), China) and positive plasmids were sequenced using Dye Terminator Cycle Sequencing ready reaction in ABI PRISM 377 DNA sequencer with v2.1.1 software according to the manufacturer’s instructions. Table 1 shows the vector primers and custom synthesized primers used for the sequencing of BoHSP90-A and BoHSP90-B genes.

In order to determine the presence of introns in BoHSP90-A and BoHSP90-B genes, the ORF of genes were amplified from cDNA and sequenced using the protocol described above.

### Sequence structure, function and motifs prediction

The obtained sequences for BoHSP90-A and BoHSP90-B genes were assembled in the Staden Package (version 1.6.0 for Windows) [21-23] and submitted to GenBank with accession numbers KF379584-KF379585. The predicted AA sequences for BoHSP90-A and BoHSP90-B were obtained by translating ORFs in ExPASy translation tool (http://web.expasy.org/translate/). The predicted structures of BoHSP90-A and BoHSP90-B were prepared using the I-TASSER Standalone Package (Version 2.1) [24] and edited in the Strap software [25]. The secondary structures and motifs of proteins were predicted by comparing with crystal structures of HSP90 of yeast (PDB code: 2cg9) [10,26] at the SWISS-MODEL server [27].

Several HSP90 inhibitors, that can alter the biological activity of BoHSP90-A and BoHSP90-B, were predicted at ProFunc server [28]. The structures of inhibitors complexed with proteins were downloaded from RCSB Protein Data Bank. The template IDs of structures of inhibitors and PDB codes of the complexed proteins have been given in Table 2. The schematic diagrams of the interaction between BoHSP90 proteins and ligands (inhibitors) were prepared and edited in the LIGPLOT^+^ software (version v.1.4.5) [29].

**Table 2:** **Interaction of *Babesia orientalis *** **HSP90-A and B isoforms with inhibitors using docking analysis**

**Name**	**Protein showing interaction**	**Template ID**	**PDB code**
Geldanamycin	BoHSP90-A, BoHSP90-B	Gmy1	2exl
Radamide	BoHSP90-A, BoHSP90-B	Rda1001	2gfd
Redicicol	BoHSP90-A, BoHSP90-B	Rdc301	1u0z
Dihydroxyphenylpyrazoles	BoHSP90-A, BoHSP90-B	4BC401	1yc1
PU8(8-(2-chloro-3,4,5-trimethoxy-benzyl)-2-fluoro-9-pent-4-ylnyl-9 h-purin-6-ylamine)	BoHSP90-B	PU11224	1uyf
PU11 (8-(2,5-dimethoxy-benzyl)-2-fluoro-9- pent-9 h-purin-6-ylamine)	BoHSP90-A	Puz1224	1uyi
1-(2-phenol)-2-naphthol	BoHSP90-A, BoHSP90-B	AB41226	2bz5
3,4-diaryl pyrazole resorcinol (CCT018159)	BoHSP90-A, BoHSP90-B	Ct51224	2bt0
4-amino derivative of 3,4-diaryl pyrazole (4-chloro-6-(4-piperazin-1-yl-1 h-pyrazol-3-yl)-benzene-1,2-diol)	BoHSP90-B	4BH1224	2ccs

The template IDs are the identification numbers of inhibitors in RCSB Protein Data Bank. The proteins complexed with the inhibitors are indicated by PBD codes.

Various antigenic peptides of BoHSP90-A and BoHSP90-B were determined using the Protean program of Lasergene^®^ software package, version 10 (DNASTAR, Madison, WI, USA). The average antigenic propensity of BoHSP90-A and BoHSP90-B was predicted based on the table of experimentally known epitopes [30].

### Recombinant expression and purification of BoHSP90-A and BoHSP90-B

**Figure 1:**
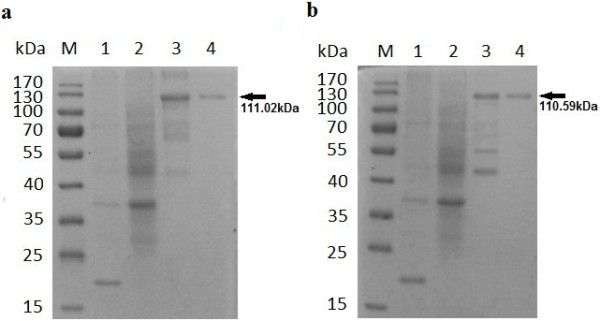
**Purification of the recombinant proteins from *E. coli *using SDS-PAGE. (a)** Purification of rBoHSP90-A. Lane M pre-stained molecular weight marker, lane 1 lysate of IPTG induced empty vector (pET-32a), 2 lysate of uninduced plasmid pET-32a/BoHSP90-A, 3 lysate of IPTG induced plasmid, 4 purified rBoHSP90-A. **(b)** Purification of rBoHSP90-B. Lane M pre-stained molecular weight marker, lane 1 lysate of IPTG induced empty vector (pET-32a), 2 lysate of uninduced plasmid pET-32a/BoHSP90-B, 3 lysate of IPTG induced plasmid, 4 purified rBoHSP90-B. The corresponding bands are indicated by arrows.

The ORFs of BoHSP90-A and BoHSP90-B were amplified from the cDNA and subcloned into the pET-32a vector using the *EcoR*I and *Xho*I restriction sites. The resulting plasmids pET-32a/BoHSP90-A and pET-32a/BoHSP90-B were transformed into the *E. coli* BL21™ (DE3) strain (Transgene, China) using the standard method. The plasmids from bacterial cells at each step were extracted using QIAGEN Plasmid Mini Kit (Qiagen, Hilden, Germany) and subjected to restriction enzyme digestion and sequencing analysis in order to verify successful cloning and the orientation of genes in the vector. The proteins were expressed in *E. coli* by inducing with 1 mM isopropyl β-D-thiogalactoside (IPTG). The rBoHSP90-A and rBoHSP90-B were expressed as His-fusion proteins and their expressions were monitored through SDS-PAGE. The uninduced plasmids pET-32a/BoHSP90-A and pET-32a/BoHSP90-B and IPTG induced empty vector (pET-32a) were used as negative controls (Figure 1).

The rBoHSP90-A and rBoHSP90-B were expressed in the insoluble-fraction of *E. coli* and were purified using HiTrap TALON crude (GE Healthcare, Sweden). The affinity chromatography was performed according to the manufacturer’s instructions. The quantities of recombinant proteins were measured by BCA protein assay kit (Beyotime Institute of Biotechnology, China) according to the manufacturer’s instructions.

### Production of anti-rBoHSP90-A and anti-rBoHSP90-B immune serum

Six Japanese white female rabbits (SPF) weighing 2 kg were divided into 2 groups (A and B). Two rabbits in each group were immunized subcutaneously with 500 μg of rBoHSP90-A and rBoHSP90-B, respectively, emulsified in equal amounts of Freund’s complete adjuvant (Sigma, USA). One untreated rabbit in groups A and B served as a negative control. The same amounts of antigens emulsified in Freund’s incomplete adjuvant (Sigma, USA) were injected subcutaneously into the immunized rabbits on days 14, 21 and 28. Two weeks after the last immunization, sera from the immunized rabbits were collected and stored at -20°C until further investigation.

### SDS-PAGE and western blotting

For the preparation of parasite lysate from infected buffalo blood, the mononuclear cells from the blood (3% parasitaemia) were removed using HISTOPAQUE^®^-1077 (Sigma, USA) according to the manufacturer’s instructions. The parasite lystae was then prepared by following the steps described elsewhere [31]. To identify native HSP90-A and HSP90-B in *B. orientalis*, the parasite lysate was subjected to 12% SDS-PAGE using the standard method followed by electroblotting onto a nitrocellulose membrane (Millipore, USA). The membranes were then probed with rabbit antisera from groups A (1:500 dilution) and B (1:500 dilution) separately and incubated overnight at 4°C. The membranes were washed 5 times with TBST and incubated with secondary antibodies (1:2000, HRP labeled goat anti-rabbit IgG, Beyotime Institute of Biotechnology) for 2 hours at room temperature. After washing, the protein bands on membranes were visualized using DAB method (ZSGB-BIO, China). The lysate of uninfected buffalo erythrocytes and the serum of untreated rabbits were used as controls.

In order to investigate the presence of antibodies in buffalo serum against BoHSP90-A and BoHSP90-B, the western blot analysis was carried out as mentioned above, but using the recombinant proteins and buffalo sera. Briefly, the rBoHSP90-A and rBoHSP90-B expressed in *E. coli* were separated on 12% SDS-PAGE. The blotted membranes were incubated with anti-*B. orientalis* buffalo immune serum (1:500) and then with secondary antibodies (1:2000, HRP labeled goat anti-bovine IgG, Sigma, USA). The serum from normal buffalo was used as a negative control. For positive control, the rBoHSP90-A and rBoHSP90-B were probed with anti-His tag antibodies (primary antibodies, 1:5000, EarthOx, LLC, USA) and then with secondary antibodies (1:2000, HRP labeled goat anti-mouse IgG, Beyotime Institute of Biotechnology, China). The antibodies at each step in the experiment were diluted with TBST containing 5% skimmed milk.

## Results

### Molecular characterization and sequence analysis

**Figure 2:**
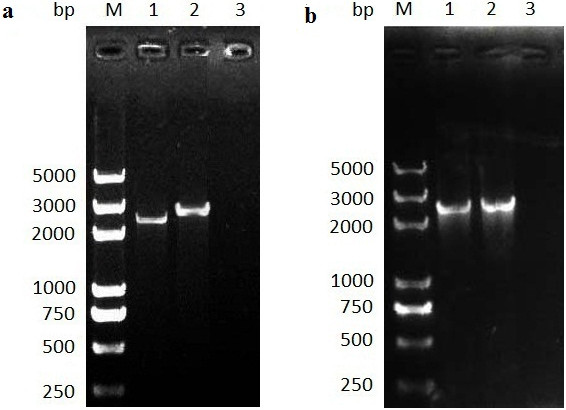
**The PCR amplification of HSP90-A and HSP90-B genes from gDNA and cDNA of *B. orientalis*. (a)** PCR products of BoHSP90-A gene. Lane M marker, lane 1 amplification from cDNA (2403 bp), 2 amplification from gDNA (2706 bp), 3 negative control. **(b)** PCR products of BoHSP90-B gene. Lane M marker, lane 1 amplification from cDNA (2394 bp), 2 amplification from gDNA (2394 bp), 3 negative control.

The HSP90-A gene of *B. orientalis* was amplified from both gDNA and cDNA by PCR using 1HSP90-F and 1HSP90-R primers, designed based on *in silico* BLAST search on *B. orientalis* genome sequences (Table 1). The amplified sequence of BoHSP90-A gene from gDNA was 2706 bp long, while, the amplicon from cDNA was 2403 bp long (Figure 2a) with complete ORF. The nucleotide sequence of BoHSP90-A gene amplified from gDNA showed the presence of one intron from position 997 to 1299 bp. The encoded polypeptide comprised of 800 AA residues with a predicted size of 91.02 kDa, as determined through a computer-based molecular weight calculator [32].

The HSP90-B gene of *B. orientalis* was amplified by PCR using 2HSP90-F and 2HSP90-R primers. The PCR amplified products from both templates were the same size (Figure 2b) and length (2394 bp), indicating no intron. The ORF encoded a polypeptide of 797 AA with a size of 90.59 kDa. The protein IDs of BoHSP90-A and BoHSP90-B in GenBank are AGY56137 and AGY56138.

The percent identity between the AA sequences of the two BoHSP90 was 27.9%. The AA sequences of BoHSP90-A and BoHSP90-B were most identical to those of *B. bovis* HSP90 (XP_001611867.1) and HSP90 putative (XP_001610762.1) genes with an identity of 86.6% and 82.2%, respectively. The percent identities between HSP90-A and HSP90-B AA sequences of *B. orientalis* and other piroplasms have been shown in the Additional file 1. The AA sequence of BoHSP90-A was well conserved in other piroplasms, showing 55-86% identity. However, BoHSP90-B AA sequence was 35-82% identical to HSP90-B sequences of other piroplasms.

### Predicted structure and functional analysis

**Figure 3:**
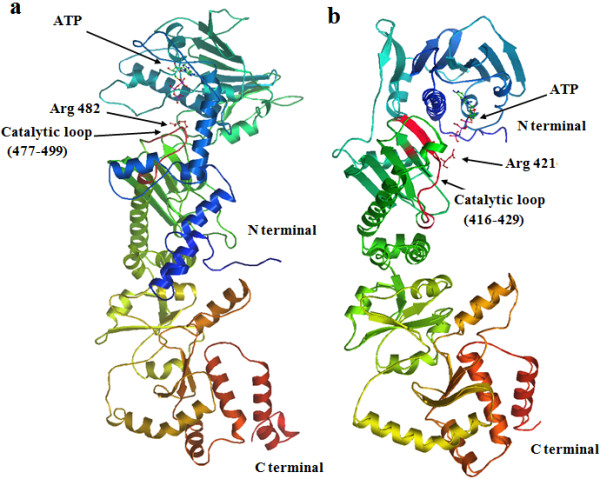
**Structural modeling of *B. orientalis *HSP90-A and B isoforms. (a)** cartoon rainbow (blue-red) of BoHSP90-A from N to C-terminal indicating the binding site for ATP and conserved Arg at position 482 in catalytic loop (red). **(b)** the structure of BoHSP90-B showing ATP binding site and conserved Arg at position 421 in catalytic loop (red).

**Figure 4:**
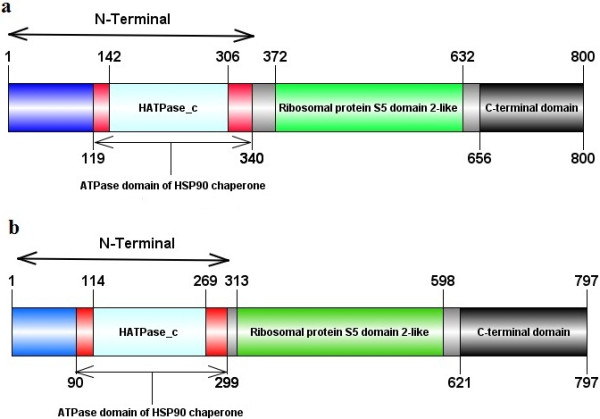
**Schematic illustrations of the predicted domains of *B. orientalis *** **HSP90-A (a) and HSP90-B (b).**

The predicted 3D structures of BoHSP90-A and BoHSP90-B were modeled through homology-modeling using the crystal structure of HSP90 of yeast (PDB code: 2cg9) [26] as a template. As shown in the Figure 3, both structures had binding sites for ATP, further confirming that these two putative proteins in *B. orientalis* belong to HSP90 family. Although, the AA sequences of BoHSP90-A and BoHSP90-B are not well conserved (27.9% identical); however, their predicted domain structures appeared to be similar (Figure 4). The predicted structure of BoHSP90-A and BoHSP90-B comprised of three domains including N-terminal, middle domain (Ribosomal protein S5 domain 2-like superfamily) and C-terminal domain (Figure 4a and b).

**Figure 5:**
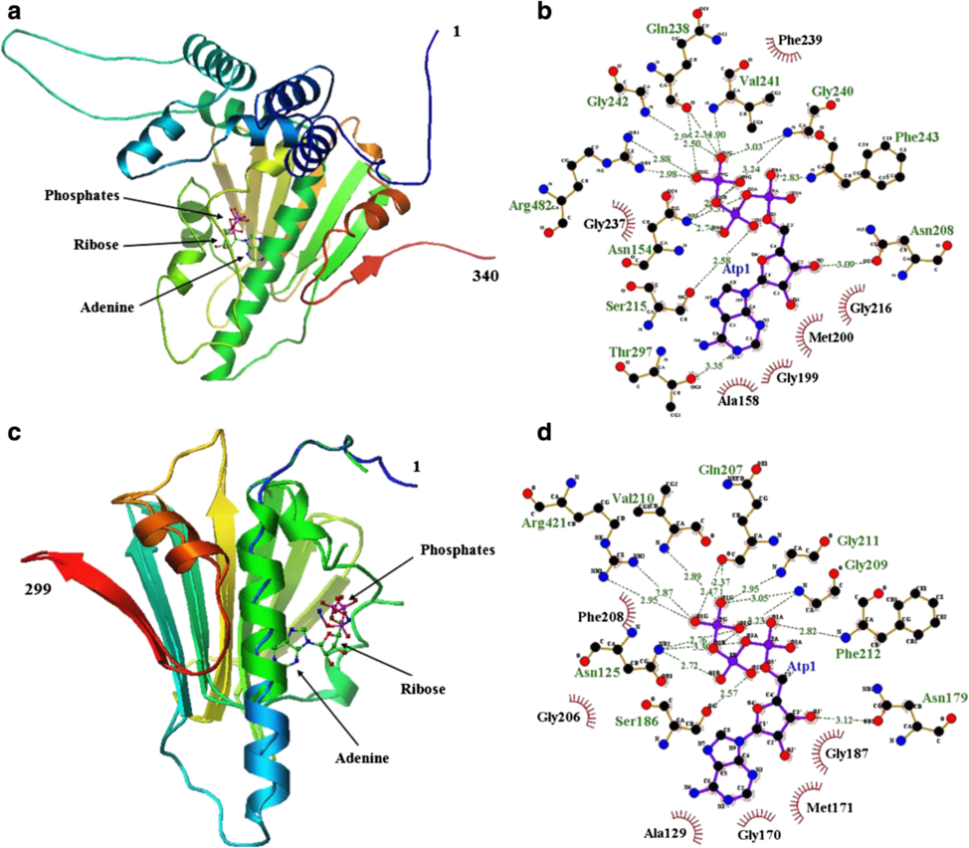
**Schematic interactions of ATP with the N-terminal domains of *B. orientalis *HSP90-A and B isoforms. (a)** and **(c)** close-up of ATP molecules bound to N-terminal domains of BoHSP90-A and BoHSP90-B, respectively. **(b)** and **(d)** biochemical interactions of ATP with the residues of BoHSP90-A and B isoforms, respectively. For key items, see Figure 7.

The N-terminal domains in BoHSP90-A and BoHSP90-B are well conserved in HSP90-A and HSP90-B of other piroplasms, respectively (Additional files 2 and 3). The N-terminal domains of the 2 putative *B. orientalis* HSP90s contained the ATP binding sites and the residues essential for ATPase activity equivalent to HATPase_c domain in MutL and type II toposimerases (Figure 5). The conserved GxxGxG motif, essential for ATP binding, in BoHSP90-A was from 237–242 residues (Figure 5b). These residues including Gly237, Gln238, Phe239, Gly240, Val241, Gly242 in BoHSP90-A were wrapping around β and γ phosphates of the ATP molecule. Among these, the residues Gly237 and Phe239 made hydrophobic interactions while the other residues along with Ser215 and Arg482 interacted with the two phosphates of the ATP through hydrogen bonds. The residues Phe243, Asn208 and Thr297 were involved in the interaction of hydrogen bonds with α phosphate, ribose sugar and adenine ring, respectively. The residues in the distal part of N-terminus were involved in hydrophobic interactions (Figure 5b).

**Figure 6:**
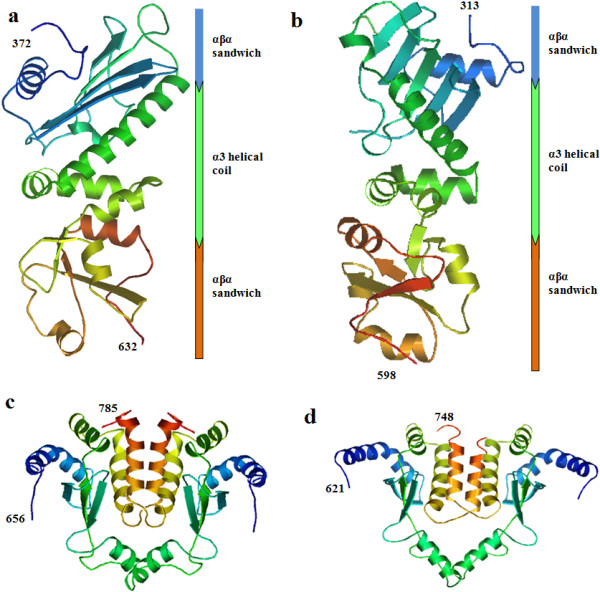
**The middle and C-terminal domains of *B. orientalis *HSP90-A and B isoforms. (a) and (b)** middle domain of BoHSP90-A and BoHSP90-B, respectively, showing three subdomains. **(c)** Dimerization interface between C-terminal domains of two BoHSP90-A molecules formed by 656–785 residues. **(d)** Dimeric C-terminal domain of BoHSP90-B formed by 621–748 residues.

In BoHSP90-B, the residues wrapping around β and γ phosphates of the ATP molecule (GxxGxG motif) were from 206 to 211, including Gly206, Gln207, Phe208, Gly209, Val210 and Gly211 (Figure 5d). Similar to BoHSP90-A, the residues Gly206 and Phe208 were involved in the hydrophobic interaction and Ser186 and Arg421 interacted through hydrogen bonds with the β and γ phosphates of ATP molecule, respectively. The α phosphate and ribose sugar of ATP molecule interacted with Phe212 and Asn179 residues of N-terminus of BoHSP90-B, respectively. In contrast, no residue of N-terminal domain of BoHSP90-B was involved in the interaction with adenine ring of the ATP molecule. However, the residues Ala129, Gly170, Met171 and Gly187 in the distal part of N-terminus were involved in the hydrophobic interactions with the adenine ring (Figure 5d). The residues in the GxxGxG motif in BoHSP90-A and BoHSP90-B are highly conserved in yeast HSP90 (residues 118–123) [10].Topologically similar to the middle domains of MutL, DNA gyrase B (Gyr B) and HSP90 proteins, the middle domains of BoHSP90-A and BoHSP90-B comprised of three distinct subdomains including a large αβα sandwich at N-terminus of middle domain, a α3 helical coil in the middle and a small αβα sandwich at the C-terminus (Figure 6a and b). All these subdomains contained essential residues for client protein binding and ATP hydrolysis. These results indicated that the middle domains of BoHSP90-A and BoHSP90-B belong to the ribosomal protein S5 domain 2-like superfamily.The hydrophobic residues in the middle domain of BoHSP90-A are on or near the Src loop (residues 427–442). Although, the residues in this loop in eukaryotes (residues 327–340 in yeast) are not well conserved in BoHSP90-A; however, the pattern of hydrophobic and hydrophilic residues is the same. Similarly, the residues 368–381 of BoHSP90-B formed a distinct Src loop, containing a combination of hydrophobic and hydrophilic residues. The middle of these two proteins also comprised a catalytic loop, which is essential for the hydrolysis of ATP. This loop in the middle domain of BoHSP90-A (residues 477–490) contains conserved Asn479, Arg482, and Gln486 (Asn377, Arg380, and Gln384 in yeast). The same residues in the catalytic loop in BoHSP90-B (residues 416–429) are at position N418, R421 and Q425, respectively. The catalytic loop in the middle domains of BoHSP90-A and BoHSP90-B along with conserved Arginine has been shown in Figure 3.

The extreme segment of the C-terminal domain of both BoHSP90-A and BoHSP90-B proteins lack the MEEVD motif, which is known to interact with tetratricopeptide repeat (TPR) domains of co-chaperones in Eukaryotes [33,34]. However, the C-terminus of BoHSP90-A and BoHSP90-B exhibited dimerization interface formed by residues 656–785 and 621–748 (correspond to 749–674 in yeast), respectively. The dimeric C-terminal domains of BoHSP90-A and BoHSP90-B have been presented in Figure 6c and d.

### HSP90 inhibitors

**Figure 7:**
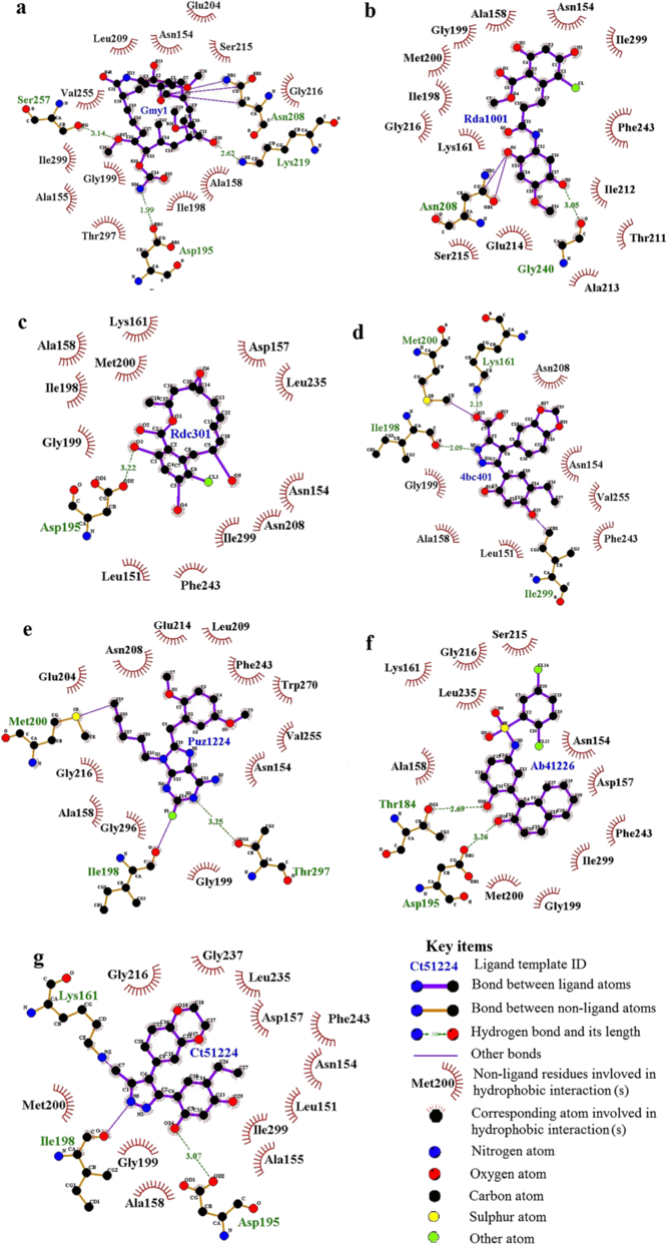
**Schematic interactions of *B. orientalis *HSP90-A isoform with its inhibitors. (a)** Geldanamycin, **(b)** Radamide, **(c)** Redicicol, **(d)** Dihydroxyphenylpyrazoles, **(e)** Purine-based derivative (PU11), **(f)** 1-(2-phenol)-2-naphthol, **(g)** 3,4-diaryl pyrazole resorcinol (CCT018159).

**Figure 8:**
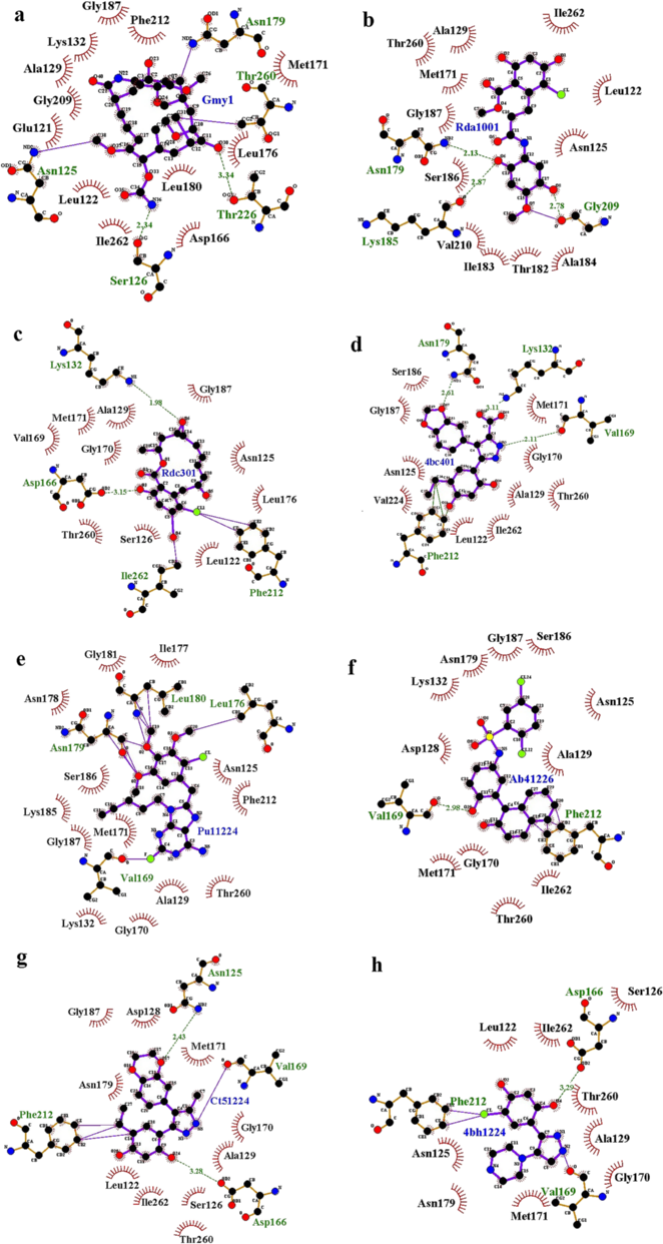
**Schematic interactions of *B. orientalis *HSP90-B isoform with its inhibitors. (a)** Geldanamycin, **(b)** Radamide, **(c)** Redicicol, **(d)** Dihydroxyphenylpyrazoles, **(e)** Purine-based derivative (PU11), **(f)** 1-(2-phenol)-2-naphthol, **(g)** 3,4-diaryl pyrazole resorcinol (CCT018159), **(h)** The 4-amino derivative of 3,4-diaryl pyrazole class.

The inhibitors of BoHSP90-A and HSP90-B identified through structure-based design include Geldanamycin (GA), Radamide (RD), Radicicol (RC), dihydroxyphenylpyrazoles, purine-based inhibitors (PU1, PU8, PU11), 4-amino derivatives (e.g. 4-chloro-6-(4-piperazin-1-yl-1 h-pyrazol-3-yl)-benzene-1,2-diol), 1-(2-phenol)-2-naphthol compounds and 3,4-diarylpyrazole class (Table 2). All these inhibitors exhibited binding to ATP binding pockets of the N-terminal domains of BoHSP90-A and BoHSP90-B.The GA-BoHSP90-A interaction analysis revealed that the residues Lys219 and Ser257 made hydrogen bonds with the methoxy group of ansa ring and ASP195 with the carbamate group. In BoHSP90-B-GA interaction, Ser126 and Thr226 interacted with carbamate and methoxy groups, respectively. A covalent bond was observed between Thr260 and methyl group, Asn125 and methoxy group and Asn179 and quinone. The residues forming hydrophobic interactions with GA are indicated in Figure 7a and Figure 8a.In RD-BoHSP90-A complex, the residues Gly240 and Asn208 interacted with quinone through hydrogen and covalent bonds, respectively (Figure 7b). The residues of BoHSP90-B interacting with the quinine of RD were Asn179, Lys185 and Gly207 (Figure 8b). The residue Asp195 of BoHSP90-A and Asp166 of BoHSP90-B made hydrogen bonds with the resorcinol ring of RC. The residues Lys132 of BoHSP90-B interacted with ethylene oxide group and Phe212 and Ile263 with the resorcinol ring of RC through covalent bonds (Figure 7c and Figure 8c).

The pyrazole NH of dihydroxyphenylpyrazole formed a hydrogen bond with the carbonyl of Ile198 of BoHSP90-A. The carboxyl group of pyrazole ring made a hydrogen bond with NH_2_ of Lys161 and CH_3_ of Met200. The residue Ile299 made a hydrogen bond with the hydroxyl group of the dihydroxyphenyl ring (Figure 7d). In dihydroxyphenylpyrazole-BoHSP90-B complex, the carbonyl of Val169 made a hydrogen bond with NH of pyrazole, NH_2_ of Lys132 with the carboxyl group of pyrazole ring, the amino group of Asn179 with oxygen of benzodioxole ring and Phe212 made polar interactions with the dihydroxyphenyl ring (Figure 8d).

Among the purine-based inhibitors, the compound 8 was observed to be the most active, inhibiting the function of human HSP90 [35]. The structure of BoHSP90-B showed binding to PU8 as investigated through computer-based drug design. The BoHSP90-B residues Lue176, Asn179 and Leu180 interacted with the three methoxy groups of the aromatic ring of PU8 through covalent bonds and hydrogen bonds. A dipole interaction was observed between Fluorine of 2-fluoro-adenine and oxygen of Val169 (Figure 8e). The structure of BoHSP90-A showed binding to compound 11 of purine-based derivatives. The hydroxyl group of Thr297 made hydrogen bond with N2 of 2-fluoro-adenine and oxygen of Ile198 made dipole interaction with Fluorine. The C5 of 1-pentynyl of PU11 interacted with the sulfur of Met200 through covalent bond (Figure 7e).Among the derivatives of 1-(2-phenol)-2-naphthol class, the structures of BoHSP90-A and BoHSP90-B complexed with compound 11. In the complex between BoHSP90-A and 1-(2-phenol)-2-naphthol, a hydrogen bond was observed between the hydroxyl group of naphthol and Asp195, and the hydroxyl group of phenol ring and Thr184 (Figure 7f). In BoHSP90-B-1-(2-phenol)-2-naphthol complex, a hydrogen bond was observed between the hydroxyl group of Phenol ring and Val169. The nonpolar benzyl group of Phe212 made covalent bonds with the benzene ring of the naphthol group (Figure 8f).The analysis of interaction between BoHSP90-A and 3,4-diaryl pyrazole resorcinol (CCT018159) revealed that the hydroxyl group of resorcinol made a hydrogen bond with side-chain oxygen of Asp195, the amino group of pyrazole with main-chain oxygen of Ile198 and the methyl group of pyrazole with main-chain amino group of Lys161 (Figure 7g). The interaction between BoHSP90-B and 3,4-diaryl pyrazole resorcinol appeared to be the same. The oxygen group of Asp166 and Val169 made hydrogen bonds with the hydroxyl group of resorcinol and the amino group of pyrazole, respectively. The amino group of Asn125 also made a hydrogen bond with the oxygen of 1,4-benzodioxane. The benzyl ring of Phe212 showed interaction with the ethyl group of resorcinol ring (Figure 8g).

The 4-amino derivatives of 3,4-diaryl pyrazole class inhibitor have also been discovered with higher efficacy against HSP90 activity [36]. The structure of BoHSP90-B showed interaction with a 4-amino derivative (4-chloro-6-(4-piperazin-1-yl-1 h-pyrazol-3- yl)-benzene-1,2-diol). However, the interactions made by the residues of BoHSP90-B with 4-amino derivatives were the same as in 3,4-diaryl pyrazole interaction. Importantly, no interaction between the residues of BoHSP90-B and piperazine group was obvious except hydrophobic interactions by Asn125, Met171 and Asn179 (Figure 8h).

### Antigenicity analysis

The antigenicity analysis revealed that the amino acid sequences of both proteins contain potential antigenic determinants. The predicted polypeptide of BoHSP90-A has 27 antigenic regions with an average antigenic propensity of 1.0250 (Additional file 4). The BoHSP90-B polypeptide contains 36 antigenic regions with an average antigenic propensity of 1.0189 (Additional file 5).

### Identification of BoHSP90-A and BoHSP90-B

The ORF of BoHSP90-A and BoHSP90-B genes were expressed as an about 111.02 kDa and 110.59 kDa in *E. coli*, respectively (Figure 1). However, the controls including uninduced plasmids (pET-32a/BoHSP90-A and pET-32a/BoHSP90-B) and IPTG induced empty vector (pET-32a) did not show any band of the expected size.

**Figure 9:**
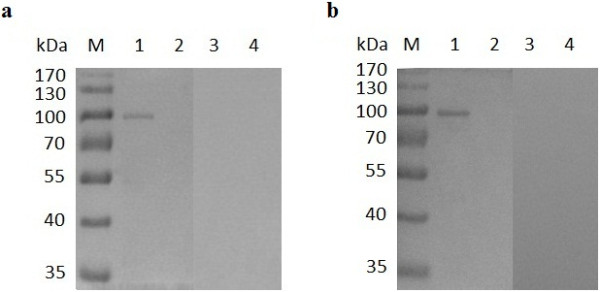
**Identification of the two putative *B. orientalis *HSP90 proteins through western blot analysis. (a)** and **(b)** the detection of BoHSP90-A and BoHSP90-B in parasite lysate, respectively. Lanes M pre-stained molecular weight markers, lanes 1 parasite lysates incubated with rabbit antisera against recombinant proteins, 2 parasite lysates incubated with serum of normal rabbit (negative controls), 3 lysates of normal buffalo erythrocytes incubated with rabbit antisera against recombinant proteins, 4 lysates of normal buffalo erythrocytes incubated with serum of normal rabbit (negative controls).

**Figure 10:**
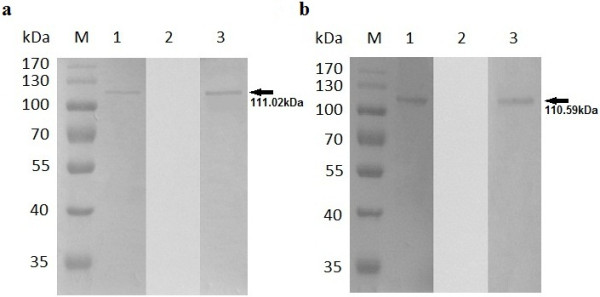
**Reactivity of *B. orientalis *recombinant HSP90 proteins with the buffalo immune serum identified through immunoblotting. (a)** and **(b)** western blot analysis of rBoHSP90-A and rBoHSP90-B, respectively. Lanes M pre-stained molecular weight markers, lanes 1 recombinant proteins reacted with buffalo immune serum, 2 recombinant proteins incubated with normal buffalo serum (negative controls), 3 recombinant proteins reacted with anti-His tag antibodies (positive controls). The arrows on the right are indicating corresponding bands.

The native HSP90-A and HSP90-B in *B. orientalis* were identified by reacting rabbit antisera with parasite lysate. As shown in Figure 9a and b, the specific strong bands corresponding to ~90 kDa proteins were obtained in *B. orientalis* lysates through western blot analysis, consistent with the predicted sizes from sequences. The controls, including the parasite lysates reacted with normal rabbit serum and the lysates of normal buffalo erythrocytes reacted with immunized rabbit and normal rabbit serum did not show any band.The sera from experimentally infected buffalo strongly reacted with rBoHSP90-A (Figure 10a) and rBoHSP90-B (Figure 10b) and recognized the specific bands of 111.02 kDa and 110.59 kDa, respectively. The bands of the same size for rBoHSP90-A and rBoHSP90-B, were recognized by anti-His tag antibodies (positive control). However, no band was obvious when the recombinant BoHSP90 proteins were reacted with the normal buffalo serum.

## Discussion

There are five functional isoforms of HSP90 in higher eukaryotes including HSP90-α1, HSP90-α2, HSP90-β, GRP-94 (Endoplasmin) and TNF Receptor-Associated Protein 1 (TRAP) [37]. In the present study, two novel isoforms of BoHSP90 encoding HSP90-A and HSP90-B were identified and characterized. Although, the analysis of full genome sequences of *Babesia* and *Theileria* revealed the presence of some HSP90 putative proteins, however, this is the first report on the molecular characterization of HSP90-A and HSP90-B in *Babesia* species. The deduced AA sequences of HSP90-A and HSP90-B of *B. orientalis* showed identity with those of *B. bovis*, *B. equi*, *Theileria parva*, *T. annulata* and *T. orientalis*. These findings indicated that the amplified products from gDNA and cDNA were HSP90-A and HSP90-B genes of *B. orientalis*.

The recognition of BoHSP90-A and BoHSP90-B from parasite lysate further confirmed that these two isoforms of HSP90 exist in *B. orientalis* as 90 kDa. A few apicomplexans and trypanosomatid protozoa have also been reported to contain a 82 kDa protein of HSP90 family including *Leishmania donovani*[11], *Trypanosoma cruzi*[12], *Toxoplasma gondii*[13], *Plasmodium falciparum*[14], *Eimeria acervulina* and *E. tenella*[15]. The structural analysis revealed that the BoHSP90-A and BoHSP90-B has various components essential for the chaperone activity. The residues essential for the ATP binding in yeast HSP90 were observed to be highly conserved in HSP90-A and HSP90-B of *B. orientalis* and other piroplasms, indicating that their N-terminal domain belongs to topoisomerase II superfamily. The BoHSP90-A and BoHSP90-B contained a glycine-rich loop (GxxGxG) within N-terminal, which wrapped around the phosphates of ATP molecule. The residues in the GxxGxG motif in yeast HSP90, including Gly118, Gln119, Phe120, Gly121, Val122, Gly123 [10], were conserved in BoHSP90-A and BoHSP90-B. This structure also overlays with the ATP binding domain of *P. falciparum*, as demonstrated in the crystal structure of N-terminal domain, in ATP bound state [38]. It has been observed that the client protein is able to bind with the middle domain of HSP90 without ATP in yeast, however, the bound ATP gives ‘tense’ conformation to HSP90 dimer which is required for the maturation of the client protein [10]. The presence of ATP binding domain along with the GxxGxG motif suggests that the functioning of BoHSP90-A and BoHSP90-B may depend on ATP hydrolysis reaction.

The crystal structures of the middle domain of HSP90 in higher eukaryotes demonstrated that the combination of hydrophobic and hydrophilic residues in the middle domain (Src loop) provides a major site for client protein binding [39]. Experimental evidence suggested that the Arg 380 in yeast interacts with γ-phosphate of an ATP molecule and is directly involved in ATP hydrolysis, which is essential for the release of client protein [10,39]. Consistent with the yeast middle domain structure, the BoHSP90-A and BoHSP90-B consist of the same pattern of hydrophobic and hydrophilic residues in the middle domain, although the residues in this loop in BoHSP90-A and BoHSP90-B are not well conserved. However, the middle domain of BoHSP90s comprises the conserved residues in the catalytic loop. Experimental evidence suggests that the catalytic loop in the middle domain of HSP90 in yeast (residues 375–388) is essential for the ATPase activity [10].

Unlike other known HSP90 structures, the C-terminal segments of two isoforms of *B. orientalis* HSP90 lack MEEVD motif, although comprising dimerization interface. The other charged domain at the extreme C-terminous segment of BoHSP90-A and BoHSP90-B may play a significant role in interaction with cochaperones. Alternatively, they may adopt a different mechanism of organizing HSP90 chaperone complex. Interestingly, this segment (MEEVD) is also lacking in the HSP90 HtpG of *E. coli*[40], this molecule was observed to be capable of client protein binding and release, as evident by crystallographic studies [41]. Alternatively, the C-terminal segment of 82 kDa homologue (HSP90) in apicomplexans contains ~30 residues ending in MEEVD motif [13,14]. The predicted structure analysis revealed that the BoHSP90-A and BoHSP90-B contain numerous domains and residues, essential for ATPase and chaperone activity. Further crystallographic studies are required to better understand the conformation of the bound ATP, client proteins and co-chaperones in HSP90 chaperone complex in *B. orientalis*.

A range of HSP90 inhibitors showed binding affinity with ATP binding pocket of BoHSP90-A and BoHSP90-B. Experimental evidence suggested that these inhibitors significantly altered the function of HSP90 in humans and have been used as anticancer drugs [42,43]. Recently, some of these ligands were used to determine their effects on parasite growth. On the other hand, the inhibition of HSP90 activity by these ligands also facilitated in exploring their function in cellular transformations during the parasite life cycle [12]. Experimental evidence suggested that the inhibition of HSP90 in protozoans significantly altered the entry of parasite into host cells and intracellular growth. GA treatment to the RH tachyzoites of *T. gondii* blocked the entry of tachyzoites into the vero cells by 75% within 4 h and significantly retarded the intracellular growth [13]. The inhibition of stage differentiation from bradyzoite to tachyzoite stage in *T. gondii* by GA is also evident [44]. Similarly, the inactivation of HSP90 by GA and RC with different sensitivities in *E. tenella*, *L. donovani*, *P. falciparum*, *T. cruzi*, *T. brucei*, and *T. evansi* has been proven [12,16-19,45]. Likewise, ATP-competitive inhibition of BoHSP90-A and BoHSP90-B may also help in investigating their roles in the invasion of parasite into the host cells and intracellular growth. The present results also provided a base line to test the effect of other HSP90 inhibitors on the function of HSP90 in apicomplexan parasites and to investigate their effect on the parasite life cycle and biology.

Surprisingly, the recombinant BoHSP90-A and BoHSP90-B were recognized by buffalo immune serum, indicating that these proteins might be secreted by parasites before the entry into host cells and they may have a significant role in the functional conformation of molecules important for invasion of sporozoites or merozoites. Likewise, the HSP70 in *B. orientalis*, involved in multi-chaperone complex in eukaryotes [46], was also recognized by buffalo immune serum [47]. The parasites during invasion are exposed to protective immunity and the environmental stress. HSP90 may play an important role in the establishment and development of these organisms [48]. Likewise, the HSP90 (82 kDa) of some protozoa have been previously detected in excretory/secretary antigens [13,16]. On the other side, the presence of antibodies against BoHSP90-A and BoHSP90-B in buffalo immune serum also indicates that these proteins may have potential antigenic sites recognized by the immune system of the host. Since, the antigenic analysis revealed the presence of potential antigenic peptides in both isoforms of BoHSP90 with an average antigenic propensity of 1, suggesting that these molecules are potential candidates for developing a subunit vaccine.

## Conclusions

The antibodies raised against recombinant BoHSP90 proteins detected these proteins in the parasite lysate as 90 kDa. The rBoHSP90-A and rBoHSP90-B were recognized by the anti-*B. orientalis* buffalo immune serum. The antigenicity analysis indicated that these molecules may have potential antigenic determinants for developing a subunit vaccine. The predicted secondary structure analysis of BoHSP90-A and BoHSP90-B revealed the presence of all domains involved in chaperonic activity, including ATP binding pocket in N-terminal domain, Src and catalytic loop in middle domain and dimerization interface in C-terminal domain. Several HSP90 inhibitors showed interaction with ATP binding pocket of BoHSP90-A and BoHSP90-B proteins. The lack of *in vitro* culture of *Babesia orientalis* was a limitation in determining the function of these proteins in parasite biology. Future experimental studies are needed to determine their function in parasite biology and their role in the invasion of parasite into host cells.

## Abbreviations

BoHSP90-A: *Babesia orientalis* heat shock protein A; BoHSP90-B: *Babesia orientalis* heat shock protein B; SPF: Specific pathogen free; gDNA: Genomic DNA; cDNA: Complementary DNA; ORF: Open reading frame; AA: Amino acid; IPTG: Isopropyl β-D-thiogalactoside; TBST: Tris-buffered saline with tween 20; HRP: Horseradish peroxidase; DAB: Diaminobenzidine; GA: Geldanamycin; RD: Radamide; RC: Radicicol; PU1: PU8, PU11, purine-based inhibitors.

## Competing interests

The author(s) declare that they have no competing interests.

## Authors’ contributions

MKK and JZ designed the study, carried out all the experiments and prepared the manuscript. LH participated in the preparation of buffalo antibodies and parasite lysate. WZ, YW and QT participated in the construction of vectors and preparation of antibodies against recombinant proteins in rabbits. QS and QY and JH were involved in the purification of recombinant proteins. YZ and RF helped to draft the manuscript, and MSS and MH were involved in revising the manuscript. All authors read and approved the final version of the manuscript.

## Supplementary Material

Additional file 1 **The percent identities between amino acid sequences of *B. orientalis *** **and other piroplasms.** Click here for file

Additional file 2 The sequence alignments of ATP binding domains of BoHSP90-A and HSP90-A of other piroplasms. Click here for file

Additional file 3 The sequence alignments of ATP binding domains of BoHSP90-B and HSP90-B of other piroplasms. Click here for file

Additional file 4 The antigenic peptides in BoHSP90-A protein. Click here for file

Additional file 5 The antigenic peptides in BoHSP90-B protein. Click here for file
