# Supplementary material for: Identification of two novel HSP90 proteins in Babesia orientalis: molecular characterization, and computational analyses of their structure, function, antigenicity and inhibitor interaction
Source: Parasit Vectors. 2014 Jun 26;7:293. doi: 10.1186/1756-3305-7-293 (PMC4089566; doi:10.1186/1756-3305-7-293)
Supplement: Additional file 1 — The percent identities between amino acid sequences of B. orientalis and other piroplasms. [file 1756-3305-7-293-S1.doc]

**Additional file 1 The percent identities between amino acid sequences of *B. orientalis* and other piroplasms.**

| **(a) Percent identity based on HSP90-A** | | | | | | |
| --- | --- | --- | --- | --- | --- | --- |
| **Species (accession number)** | **1** | **2** | **3** | **4** | **5** | **6** |
| 1. *B. orientalis* (AGY56137) |  |  |  |  |  |  |
| 1. *B. bovis* (XP_001611867.1) | 86.6 |  |  |  |  |  |
| 1. *B. equi* (XP_004830928.1) | 68.8 | 70.0 |  |  |  |  |
| 1. *T. parva* (XP_764281.1) | 55.7 | 56.0 | 57.2 |  |  |  |
| 1. *T. annulata* (XP_953286.1) | 55.4 | 55.8 | 57.2 | 87.6 |  |  |
| 1. *T. orientalis* (BAM41905.1) | 56.6 | 56.6 | 57.9 | 69.6 | 68.9 |  |
| **(b) Percent identity based on HSP90-B** | | | | | | |
| 1. *B. orientalis* (AGY56138) |  |  |  |  |  |  |
| 1. *B. bovis* (XP_001610762.1) | 82.2 |  |  |  |  |  |
| 1. *B. equi* (XP_004833528.1) | 52.7 | 54.2 |  |  |  |  |
| 1. *T. parva* (XP_766455.1) | 38.4 | 38.0 | 43.6 |  |  |  |
| 1. *T. annulata* (XP_953842.1) | 38.7 | 38.1 | 44.9 | 77.2 |  |  |
| 1. *T. orientalis* (BAM39425.1) | 36.5 | 35.5 | 41.3 | 66.4 | 65.3 |  |
