# Supplementary material for: Identification of two novel HSP90 proteins in Babesia orientalis: molecular characterization, and computational analyses of their structure, function, antigenicity and inhibitor interaction
Source: Parasit Vectors. 2014 Jun 26;7:293. doi: 10.1186/1756-3305-7-293 (PMC4089566; doi:10.1186/1756-3305-7-293)
Supplement: Additional file 2 — The sequence alignments of ATP binding domains of BoHSP90-A and HSP90-A of other piroplasms. [file 1756-3305-7-293-S2.doc]

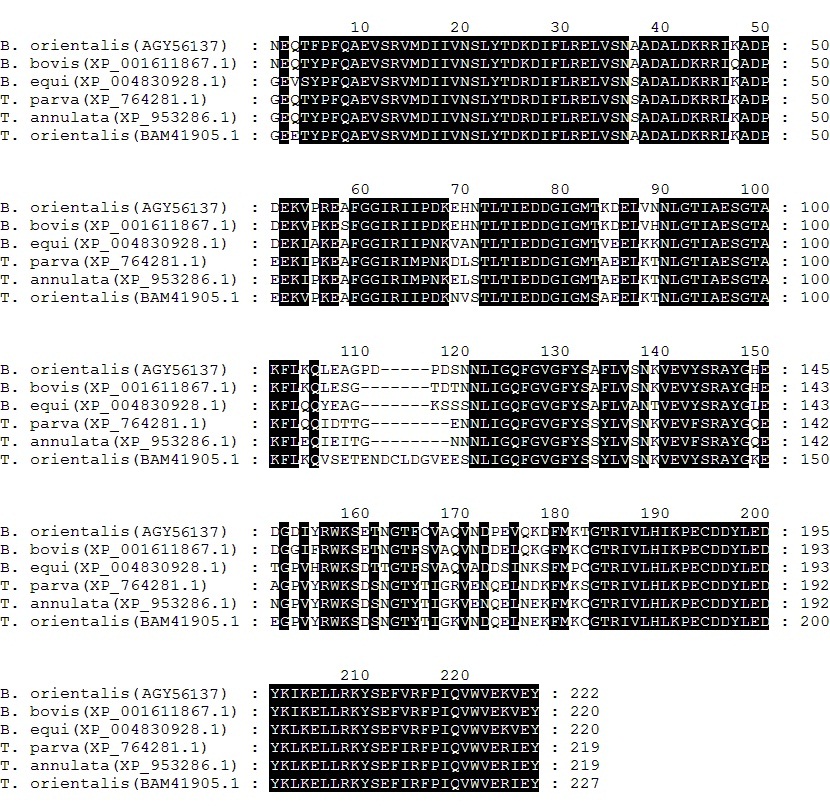


**Additional file 2** The sequence alignments of ATP binding domains of BoHSP90-A and HSP90-A of other piroplasms.
