# Supplementary material for: Identification of two novel HSP90 proteins in Babesia orientalis: molecular characterization, and computational analyses of their structure, function, antigenicity and inhibitor interaction
Source: Parasit Vectors. 2014 Jun 26;7:293. doi: 10.1186/1756-3305-7-293 (PMC4089566; doi:10.1186/1756-3305-7-293)
Supplement: Additional file 4 — The antigenic peptides in BoHSP90-A protein. [file 1756-3305-7-293-S4.doc]

**Additional file 4 The antigenic peptides in BoHSP90-A protein**

| **n** | **Start Position** | **Sequence** | **End Position** |
| --- | --- | --- | --- |
| 1 | 6 | ALRGVQA | 12 |
| 2 | 14 | RWTFYVLLAALCIYS | 28 |
| 3 | 38 | DIGSLHPRL | 46 |
| 4 | 58 | TSRPLYGF | 65 |
| 5 | 110 | SPEPQVKV | 117 |
| 6 | 122 | TFPFQAEVSRVMDIIVNS | 139 |
| 7 | 144 | KDIFLRELVSNA | 155 |
| 8 | 217 | TAKFLKQL | 224 |
| 9 | 236 | IGQFGVGFYSAFLVSNKVEVYSR | 258 |
| 10 | 276 | GTFCVAQVND | 285 |
| 11 | 296 | GTRIVLHIKPECDDYLED | 313 |
| 12 | 315 | KIKELLRKYSEFVRFPIQVWVEK | 337 |
| 13 | 382 | ADVKPEDYVSFYKSTF | 397 |
| 14 | 399 | AYDDPLSYIHFKVEGQVEFSCLLFVPGSLPW | 429 |
| 15 | 441 | RGIRLYVKRVFI | 452 |
| 16 | 456 | FSEAVPRWLTFVRGVVDSDELALNVGR | 482 |
| 17 | 484 | YLQRSKALTI | 493 |
| 18 | 523 | HFGKYIKIGVV | 533 |
| 19 | 541 | DLASLVT | 547 |
| 20 | 570 | KQPAIYYL | 577 |
| 21 | 584 | AAQSSPSLEKLKALDYEVLYALEPVDEFCFSSLTA | 618 |
| 22 | 622 | KNIMVLDVNKSD | 633 |
| 23 | 653 | EYETLCSWLKQLFPDDVHDVKISKRLVESPAILVQTDFGLSP | 694 |
| 24 | 719 | MVSKPVLEINVDHPIIQHLNLMVKA | 743 |
| 25 | 745 | KLSDVPRQVAKQLLDVVSI | 763 |
| 26 | 770 | KNPTLFAKNVLQL | 782 |
| 27 | 787 | AKQFLEKQS | 795 |
