# Supplementary material for: Identification of two novel HSP90 proteins in Babesia orientalis: molecular characterization, and computational analyses of their structure, function, antigenicity and inhibitor interaction
Source: Parasit Vectors. 2014 Jun 26;7:293. doi: 10.1186/1756-3305-7-293 (PMC4089566; doi:10.1186/1756-3305-7-293)
Supplement: Additional file 5 — The antigenic peptides in BoHSP90-B protein. [file 1756-3305-7-293-S5.doc]

**Additional file 5 The antigenic peptides in BoHSP90-B protein**

| **n** | **Start Position** | **Sequence** | **End Position** |
| --- | --- | --- | --- |
| 1 | 4 | SIFFILQCVAAISAIN | 19 |
| 2 | 21 | IQNGVVATDADLVDEPILDI | 40 |
| 3 | 46 | KEVPLDV | 52 |
| 4 | 65 | SDEVVIT | 71 |
| 5 | 73 | PEDLVSL | 79 |
| 6 | 94 | HTYQADF | 100 |
| 7 | 102 | RVMDIIVNS | 110 |
| 8 | 113 | SNKEVFLRE | 121 |
| 9 | 128 | DALEKYKIVE | 137 |
| 10 | 149 | LAIRIRV | 155 |
| 11 | 160 | RTLTILD | 166 |
| 12 | 204 | LIGQFGVGFYSVFLVADSVVVQT | 226 |
| 13 | 230 | DDKQYVWK | 237 |
| 14 | 260 | TQITLFL | 266 |
| 15 | 279 | IEELIKKHSQFVRFPIYVLK | 298 |
| 16 | 303 | AGEAVWHHVNDVKP | 316 |
| 17 | 331 | NAFYKAIS | 338 |
| 18 | 341 | HSNPLAHIHFVAE | 353 |
| 19 | 356 | VDFRALLFIP | 365 |
| 20 | 378 | VGHQVKIYARRVLVSDSLPNFLPRYLYSLYGVVDS | 412 |
| 21 | 415 | FPLNVSREHLQQ | 426 |
| 22 | 428 | KMIKIIGKKIVRSVLGT | 444 |
| 23 | 494 | GSLKVACYD | 502 |
| 24 | 509 | KISKLLMY | 516 |
| 25 | 527 | TLDQYVA | 533 |
| 26 | 539 | QKAIYYAS | 546 |
| 27 | 550 | YDAIHNSPHLQ | 560 |
| 28 | 566 | NIDVIYL | 572 |
| 29 | 577 | DESCITQ | 583 |
| 30 | 591 | SFKSVQKG | 598 |
| 31 | 616 | LKKYAPLIKAFKQHVNEIYDVKLSH | 640 |
| 32 | 643 | TEDPCTVVVS | 652 |
| 33 | 660 | MEKIVKSYIV | 669 |
| 34 | 712 | NFVDSIKLLYNAAKL | 726 |
| 35 | 737 | MLSQTAYAYLSDQLRVDS | 754 |
| 36 | 757 | TIDDIPY | 763 |
